# Case Series of Synthetic Cannabinoid Intoxication from One Toxicology Center

**DOI:** 10.5811/westjem.2016.2.29519

**Published:** 2016-04-26

**Authors:** Kenneth D. Katz, Adam L. Leonetti, Blake C. Bailey, Ryan M. Surmaitis, Eric R. Eustice, Sherri Kacinko, Scott M. Wheatley

**Affiliations:** * Department of Emergency Medicine, Lehigh Valley Health Network, Allentown, Pennsylvania; † NMS Labs, Willow Grove, Pennsylvania; ‡ Pediatric Critical Care, Lehigh Valley Health Network, Allentown, Pennsylvania

## Abstract

Synthetic cannabinoid use has risen at alarming rates. This case series describes 11 patients exposed to the synthetic cannabinoid, MAB-CHMINACA who presented to an emergency department with life-threatening toxicity including obtundation, severe agitation, seizures and death. All patients required sedatives for agitation, nine required endotracheal intubation, three experienced seizures, and one developed hyperthermia. One developed anoxic brain injury, rhabdomyolysis and died. A significant number were pediatric patients. The mainstay of treatment was aggressive sedation and respiratory support. Synthetic cannabinoids pose a major public health risk. Emergency physicians must be aware of their clinical presentation, diagnosis and treatment.

## INTRODUCTION

Synthetic cannabinoids (SCs) were synthesized to mimic the effects of Δ-9 Tetrahydrocannabinol (THC), the psychoactive component of the Cannabis sativa plant. Due to excessive cannabinoid receptor 1 (CB_1_) and cannabinoid receptor 2 (CB_2_) receptor agonism, use of SCs has been associated with unexpected and significant toxicity, including lethargy, agitation, tachycardia, hyperthermia, acute tubular necrosis, myocardial infarction, seizure and even death.^1^ Marketed as “Spice” and “K2,” its popularity among adolescents and adults has risen substantially and is the second most common drug of abuse (after marijuana) among high school students.^2^

In April 2015, an unprecedented, massive nationwide surge in SC cases was reported to regional poison control centers. The American Association of Poison Control Centers reported a 562% increase in April compared to March (1,512 versus 269).^3^ The state of New York issued a health alert after more than 160 citizens were hospitalized between April 8–17 for suspected SC toxicity. Health departments in Alabama, Mississippi, Connecticut, Maryland and the National Institute on Drug Abuse issued similar warnings.^4^ Tyndall et al. recently reported an outbreak of MAB (or ADB)-CHMINACA toxicity in multiple patients at the University of Florida Health Medical Center.^5^ Northeastern Pennsylvania simultaneously witnessed an epidemic of SC exposures.

The following cases describe 11 patients presenting to a tertiary care medical facility between April 20 and June 6 of 2015, who had serologic confirmed exposure to a novel, carboxamide indazole SC, MAB-CHMINACA (N-(1-amino-3,3-dimethyl-1-oxobutan-2-yl)-1-(cyclohexylmethyl)-1 H-indazole-3-carboxamide).^6^ This case series received expedited approval from the hospital’s institutional review board.

## CASE REPORTS

Case #1: An 18-year-old man without prior medical history was found by police, unresponsive in a parking lot after smoking “K2.” The patient became agitated and was brought into the emergency department (ED). He was tachycardic and admitted to the tertiary care intensive care unit (TCICU). His EKG showed sinus tachycardia and rightward axis. Pupil examination was 4mm bilaterally with sluggish reactivity. The patient recovered uneventfully and was discharged later that day.

Case #2: A 28-year-old man with a history of substance abuse and hepatitis presented to an outside healthcare facility (OHF) unresponsive, hallucinating and tachycardic. The patient was endotracheally intubated for airway protection and then transferred to a TCICU. Although he developed aspiration pneumonia, he was treated and discharged uneventfully on hospital day (HD) six.

Case #3: A 17-year-old woman without medical history was transferred to the TCICU from an OHF for agitation, delirium and tachycardia after exposure to SC. She was treated with benzodiazepines, but did not require intubation. he patient was discharged uneventfully on HD two.

Case #4: A 14-year-old boy without medical history was transferred from an OHF to the tertiary care pediatric intensive care unit (TCPICU) after being found unresponsive on the street. The patient became agitated and combative at the OHF, and was endotracheally intubated. The patient admitted to using “K2” and was eventually extubated and discharged on HD two.

Case #5: A 13-year-old girl with a history of marijuana abuse was transferred from an OHF to a TCPICU after ingesting SC. The patient was found intermittently responsive and combative at home. Her pupils were 3mm bilaterally and sluggish, but reactive. At the OHF, she was tachycardic and--after being administered benzodiazepines--became obtunded with hypoventilation. She was endotracheally intubated and transferred to the TCPICU. The patient was extubated and discharged on HD two.

Case #6: A 13-year-old boy without medical history was found unresponsive in a park. On examination, his pupils were 3mm bilaterally and reactive. At the OHF, the patient was endotracheally intubated and transferred to a TCPICU. He required significant amounts of sedatives due to periods of agitation and combativeness. During his hospitalization the patient developed aspiration pneumonia, but was discharged uneventfully on HD three.

Case #7: A 50-year-old man with a history of polysubstance abuse was transported to the ED after using SCs and being found unresponsive by his roommate. The patient was apneic and cyanotic, and--after endotracheal intubation--was transferred to the TCICU. He was extubated, and discharged to an inpatient drug and rehabilitation facility on HD eight.

Case #8: A 40-year-old woman with a history of bipolar disorder presented to the ED with a witnessed seizure after smoking SC. Her EKG demonstrated sinus tachycardia with an incomplete right bundle branch block. The patient was tachycardic, administered benzodiazepines and endotracheally intubated. In the TCICU, the patient was extubated, had an unremarkable electroencephalogram and discharged on HD two.

Case #9: A 19-year-old woman with a history of epilepsy, bipolar disorder and substance abuse presented to an OHF after suffering a seizure after smoking SC. Her EKG showed sinus rhythm with sinus arrhythmia and nonspecific T-wave abnormality. The patient was found unresponsive; she was endotracheally intubated and transferred to a TCICU. She was also treated for a wound infection secondary to intravenous drug abuse. The patient was extubated and discharged on HD four.

Case #10: A 14-year-old boy with a history of substance abuse was found by his mother, agitated after exposure to SC and transported to an OHF. His pupils were 4mm bilaterally and sluggishly reactive on examination. The patient’s EKG demonstrated sinus bradycardia; early repolarization was noted. The patient demonstrated periods of unresponsiveness followed by severe agitation and combativeness requiring sedation and endotracheal intubation. He was transferred to the TCICU and eventually extubated and discharged on HD two.

Case #11: A 20-year-old man without medical history was found unresponsive by family members after SC exposure and transported to an OHF. The patient had been without medical care for approximately 24 to 36 hours. He was hyperthermic, tachycardic and demonstrated decorticate posturing along with areas of trunk and extremity edema on examination. He was endotracheally intubated. Laboratory analysis demonstrated significant rhabdomyolysis and acute renal failure. He was transferred to the TCICU where clinical examination was consistent with anoxic brain injury. A brain magnetic resonance imaging (MRI) scan confirmed these findings. Care was eventually withdrawn due to the grim prognosis, and the patient died on HD seven.

These 11 patients were all exposed to the SC, MAB-CHMINACA, and presented to the ED with significant, life-threatening toxicity, ranging from obtundation to severe agitation, seizures and death. All had serum samples drawn upon arrival to the hospital that were sent to a contracted laboratory for further identification of specific SCs. All patients had expanded toxicologic analysis (liquid chromatography/mass spectroscopy, LC/MS) to determine any potential adulterants or other substances to which each patient was exposed. All patients required benzodiazepines or other sedatives for agitation. Nine of 11 patients required endotracheal intubation for either control of severe agitation or decreased responsiveness. Three experienced seizures. One patient presented with hyperthermia, and six of the 11 had tachycardia. One patient developed rhabdomyolysis and died. The majority suffered from mental illness or substance abuse. A significant number were pediatric patients, the youngest of whom was only 13. Common chemistries and laboratory values were unremarkable for these patients; pupil size examination and/or EKG results were not available for five of the cases. The results are summarized in Table.

Any drug material recovered from patients was analyzed by the county criminal investigative laboratories to determine the presence of SCs (see Figure). Results confirmed MAB-CHMINACA in all samples tested.

## DISCUSSION

To date, this is the largest SC case series describing MAB-CHMINACA toxicity. In the fall of 2014, MAB-CHMINACA was responsible for more than 125 patients seeking hospital care in Baton Rouge, LA.^7^ It is a highly potent SC, which was just recently added to the Schedule 1 Controlled Substance Act in late 2015.^8^ The clinical manifestations observed in these patients appear similar to those described with other SC toxicities.^5^,^9^

The exact mechanism of MAB-CHMINACA toxicity is unknown. Affinities of SCs and their metabolites are multifold higher at the CB_1_ and CB_2_ receptors than that of Δ-9 THC and are thought responsible for such severe clinical manifestations.^9^ CB_1_ receptors are mostly located in the brain and regulate the central nervous system effects of Δ-9 THC and other cannabinoids; they are also expressed peripherally in adipocytes and skeletal muscle.^1^ The identification of CB_1_ receptors presynaptically on GABA and glutamatergic terminals with increased excitatory and decreased inhibitory tone may be responsible.^10^ Other postulated mechanisms include activation of non-cannabinoid receptors and drug-drug synergistic effects.^1^

Some patients demonstrated stimulant and serotonergic agent use as detected by comprehensive urine drug LC/MS testing that could have contributed to their clinical presentation, such as sympathomimetic or serotonergic toxidromes. However, this was not universal and would not explain the fairly consistent clinical presentation of all of these patients. Further, the relative concentrations of MAB-CHMINACA do not necessarily correlate with toxicity and may demonstrate the potential lipophilic distribution of the drug to tissue at the time of blood draw. For example, patient #11 had a relatively low concentration, yet expired. Interestingly, post-mortem analysis of a patient who died from MAB-CHMINACA toxicity demonstrated a relatively low amount in the adipose tissue, atypical of other SCs. The authors hypothesized the compound may require time to distribute to that tissue, and that may have been the salient issue regarding patient #11 given the length of time from medical attention.^11^

Limitations in real-time detection by routine toxicologic immunoassay screening appear to be a factor in SC use.^12^ The reasons for the abrupt, recent increase in exposures, however, remain unclear. The celebration of “4/20” (a counterculture holiday celebrating Cannabis) correlated with this surge and might have influenced users.

Management of the SC-intoxicated patient centers on meticulous supportive care, as no specific antidote currently exists. Decontamination is of little value. Given their beneficial pharmacokinetic profile, administration of benzodiazepines titrated to clinical effect can control agitation, delirium, hyperthermia and seizures. Cooling measures without antipyretic administration can also be implemented for hyperthermic patients. Administration of intravenous fluids is helpful for associated rhabdomyolysis. Endotracheal intubation may be required for the severely intoxicated patient.^9^

## CONCLUSION

SCs pose a substantial and dangerous public health risk. Emergency physicians must be aware of their clinical presentation, difficulties in identification and treatment. Further studies are required to elucidate the exact mechanism of SCs and, more specifically, MAB-CHMINACA toxicity.

## Figures and Tables

**Figure:**
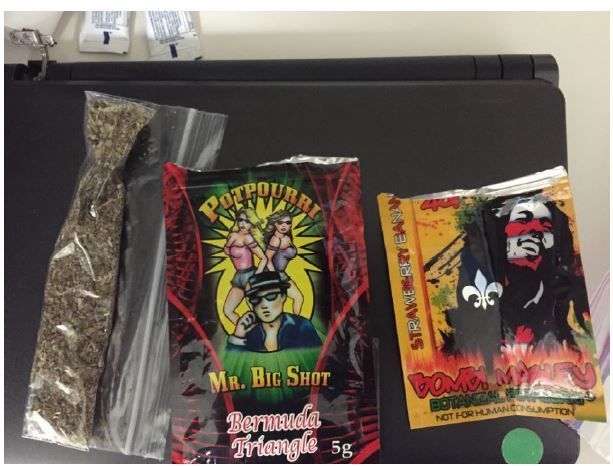
Sample synthetic cannabinoid packaging.

**Table:** Summary of case reports of 11 patients presenting to the emergency department with life-threatening toxicity after exposure to synthetic cannabinoids.

Case	Hospital day	Clinical presentation	Intubated	Outcome	Vital signs	Qualitative serum level, SC screen*	Comprehensive urine drug screen findings (LC/MS)
#1. 18yo male	Day 1	Altered mental status, combative, agitated	No	Discharged same day.	HR 113bpm, BP 95/53mmHg, temp 99.9°F, RR 20rpm, pulse ox 100%, room air	X1	Caffeine
#2. 28yo male	Day 3	Combative and hallucinating	Yes	Developed aspiration pneumonia. Discharged hospital day 6.	HR 102bpm, BP 119/88mmHg, temp 96°F, RR 15rpm, pulse ox 100%, vent	X10	Caffeine, morphine, midazolam, lorazepam
#3. 17yo female	Day 2	Agitated and delirious	No	Discharged following day.	HR 110bpm, BP 112/63mmHg, temp 97.3°F, RR 16rpm, pulse ox 98%, room air	X8	Lorazepam
#4. 14yo male	Day 2	Intermittent unresponsiveness followed by agitation	Yes	Discharged following day.	HR 96bpm, BP 123/58mmHg, temp 96.7°F, RR 19rpm, pulse ox 98%, vent	X45	Norfentanyl
#5. 13yo female	Day 2	Altered mental status with unresponsiveness	Yes	Discharged hospital day 2.	HR 115bpm, BP 98/46mmHg, temp 98.9°F, RR 17rpm, Ppulse ox 100%, vent	X20	Phenylephrine, midazolam, fentanyl, norfentanyl, diphenhydramine, cotinine
#6. 13yo male	Day 0	Found unresponsive	Yes	Developed aspiration pneumonia. Discharged on hospital day 3.	HR 81bpm, BP 131/80mmHg, temp 97.1°F, RR 16rpm, pulse ox 99%, room air	X30	Lorazapam, hydroxymidazolam
#7. 50yo male	Day 1	Found unresponsive	Yes	Discharged to inpatient drug rehabilitation facility.	HR 59bpm, BP 93/57mmHg (patient prescribed Lopressor), temp 98.7°F, RR 14rpm, pulse ox 95%, vent	X36	Ethanol, naloxone, metoprolol, caffeine
#8. 40yo female	Day 1	Combative, delirious, seizures	Yes	Discharged hospital day 2.	HR 114bpm, BP 120/62mmHg, temp 99.4°F, RR 18rpm, pulse ox 84%, room air (improved after intubation)	X32	Acetaminophen
#9. 19yo female	Day 3	Found unresponsive; seizure-like activity	Yes	Treated for infected right forearm wound secondary to IV drug use. Discharged hospital day 4.	HR 74bpm, BP 118/85mmHg, temp 97.6°F, RR 14rpm, pulse ox 100%, 40% oxygen	X55	Morphine, norfentanyl, cocaine, amphetamine, methamphetamine, codeine, midazolam, lorazepam
#10. 14yo male	Day 2	Agitated and combative	Yes	Discharged following day.	HR 95bpm, BP 114/66mmHg, temp 99.9°F, RR 22rpm, pulse ox 100% vent	X13	Sertraline
#11. 20yo male	Day 3	Found unresponsive and posturing; approximate down-time was 24–36 hours	Yes	Expired. Anoxic brain injury. Family withdrew care on hospital day 7	HR 146bpm, BP 200/74mmHg, temp 104.1°F, RR 33rpm, pulse ox 100%, vent (outside facility) HR 75bpm, BP 138/55mmHg, temp 99.5°F, RR 17rpm, pulse ox 100%, vent (upon ICU arrival)	X1.5	Sertraline

*SC*, synthetic cannabinoids; *LC*, liquid chromatography; *MS*, mass spectroscopy; *HR*, heart rate; *BP*, blood pressure; *RR*, respiratory rate; *RPM*, respirations per minute; *yo*, year old

* Value represents the number of times higher each case was in comparison to Case #1, which had the lowest relative concentration.

*SC,* synthetic cannabinoids; *LC*, liquid chromatography; *MS*, mass spectroscopy; *IV*, intravenous; *HR*, heart rate; *BP*, blood pressure; *RR*, respiratory rate; *RPM*, respirations per minute; *ICU,* intensive care unit
